# Effective Adsorption of Colorants from Sugarcane Juice by Bagasse-Based Biochar-Hydroxyapatite Composite

**DOI:** 10.3390/foods11142171

**Published:** 2022-07-21

**Authors:** Cheng Wang, Mengying Luo, Caifeng Xie, Kai Li, Fangxue Hang, Changrong Shi, William O. S. Doherty

**Affiliations:** 1 College of Light Industry and Food Engineering, Guangxi University, Nanning 530004, China; jonas_cheng_w@163.com (C.W.); luomengyinggxu@163.com (M.L.); fcx11@163.com (C.X.); gxlikai@gxu.edu.cn (K.L.); 2 Provincial and Ministerial Collaborative Innovation Center for Sugar Industry, Nanning 530004, China; 3 Engineering Research Center for Sugar Industry and Comprehensive Utilization, Ministry of Education, Nanning 530004, China; 4 Centre for Agriculture and the Bioeconomy, Faculty of Science, Queensland University of Technology, Brisbane, QLD 4000, Australia; w.doherty@qut.edu.au; 5 School of Mechanical, Medical and Process Engineering, Faculty of Engineering, Queensland University of Technology, Brisbane, QLD 4000, Australia

**Keywords:** adsorption, bagasse, colorant, decolorization, sugarcane juice

## Abstract

The clarification of sugarcane juice is a crucial stage in the sugar manufacturing process, as it affects evaporator performance, sugar quality and yield. The emergence of environmentally friendly and efficient adsorption technology has resulted in widespread interest in carbon-based materials. However, their low adsorption capacity and reusability make them unsuitable for processing sugarcane juice. Here, we provide a cost-effective and sustainable method to dope hydroxyapatite (HAP) nanoparticles on porous carbon (BBC) derived from sugarcane bagasse (BBC-HAP). The composite shows excellent adsorption capacity for color extract from sugarcane juice of 313.33 mg/g, far more effective than the commercially available carbon-based adsorbents. Isotherm studies show that the adsorption of BBC-HAP composite to the colorants is a monolayer process. The pseudo-first-order (PFO) and pseudo-second-order (PSO) kinetic models demonstrate that the adsorption process is dominated by chemisorption and supplemented by physical adsorption.

## 1. Introduction

The production of sugar from sugarcane juice is a multi-stage process [1]. It usually includes juice extraction, juice clarification, evaporation, sucrose crystallization, separation and drying [2]. Clarification of sugarcane juice is crucial in the manufacturing of sugar as it removes non-sucrose impurities and color to improve the juice purity, and hence increase the yield and quality of sugar produced. The clarification technologies of sugarcane juice such as defecation, phosphatation, sulfitation, and carbonatation, though effective, are not effective enough to remove flavonoids and phenolics (except the sulfitation process). However, the use of sulfur dioxide causes pollution to the environment and residual sulfur in the white sugar product is harmful to human health. Furthermore, the color removal effectiveness from the use of sulfur dioxide is temporary, as the product sugar develop color with storage time. The carbonatation process [3], though less effective than the sulfitation method, results in the excess carbonate ending up in the filter mud and cannot be recovered. Ion exchange resins [4] can be used but need to be regenerated to reduce costs, but the main problem with their use is the difficulty of managing the large amount of wastewater generated, and reducing the use of chemical additives to reduce the environmental impact and increase the safety of sugar is in line with the needs of social development. The benefits of membrane filtration for sugarcane juice streams have been demonstrated as it significantly reduces juice color and color precursors and reduces low and high molecular weight proteins and polysaccharides [5]. The reduction of these macromolecules reduces the viscosity of the syrup, thus allowing the production of higher quality and lower color sugars, but the current study found high membrane clear juice color values, for the reasons analyzed, yet still more lime is added in the use of the membrane and the maintenance and operation costs are high [6]. Therefore, research for a cost-effective technology to remove juice color and to minimize costs during syrup decolorization is required.

Adsorption is a popular green separation method because of its simple design, cheap cost, ease of maintenance, and great efficiency [7,8]. One of the most promising adsorbents are porous carbon compounds because of their unmatched porous structure, economic suitability and environmental friendliness [9,10]. Bagasse is a major industrial by-product of the sugar manufacturing process with ~700 million tons per year produced [11]. The majority of bagasse is burned or wasted, causing environmental issues. As a major carbon source, it has a high carbon content and is a useful resource for making biochar [12]. However, the low surface area of traditional carbon materials and the lack of highly active adsorption sites on the carbon surface have hampered the practical applicability of biological carbon materials [13]. Previous research has found that the adsorption capacity of biological carbon materials is directly proportional to the richness of their hierarchical pore structure [14]. As a result, the carbon body alone cannot solve the issue of low adsorption capacity [15]. In this case, expanding the specific surface area of the material and further increasing the effective adsorption sites of biochar is a key approach.

Hydroxyapatite (HAP) is a non-toxic, highly biocompatible, low water soluble, low-cost nanomaterial that exhibits strong adsorption capacity for various impurities in aqueous media and is considered as an environmentally friendly adsorbent for the long-term treatment of pollutants [16]. A minor fraction of HAP is generated with amorphous calcium phosphate during the defecation or phosphatation of sugarcane juice, both of which remove contaminants and color from the juice. Despite these benefits, the strong surface reactivity of HAP nanoparticles leads to the easy formation of aggregates in aqueous solutions due to van der Waals forces, reducing their dispersion in solution as well as their own surface area, which ultimately leads to their reduced adsorption performance on target contaminants [17,18,19]. The combination of high surface area biocarbon and HAP nanoparticles can effectively prevent HAP nanoparticles from self-agglomeration by dispersing HAP into the porous material. This will improve adsorption efficiency by increasing the active adsorption sites on the carbon surfaces. Therefore, using bagasse-based carbon and HAP in a composite is a promising adsorbent to improve color removal in sugarcane juice during the clarification stage. The membrane clear juice is different from the general clarified juice, and the preparation of BBC-HAP adsorbent and the study of its sugar juice decolorization process is valuable and meaningful when used in combination with membrane separation technology, which can contribute to the development of a green sugarcane sugar production technology. Real cane juice colorants extracted from membrane clear juice were used to study the adsorption capacity of biochar and hydroxyapatite composite adsorbents and the results compared to commercial adsorbents. Adsorption isotherms (Langmuir and Freundlich) and kinetic models (pseudo-first-order, PFO; pseudo-second-order, PSO; and pseudo-first-order combined with pseudo-second-order, PFO-PSO) were used to investigate the mechanism of action of the designed composites and to reveal the mechanistic pathways of adsorption.

## 2. Materials and Methods

The bagasse came from the Guangxi Fengshan sugar factory, Shanghai, China. All chemicals/reagents were analytical reagents (AR grade). Phosphoric acid, calcium nitrate tetrahydrate, diamine hydrogen phosphate, ammonia, sodium hydroxide, and hydrochloric acid were purchased from the Shanghai Sinopharm Company, Shanghai, China.

The specific chemical bonds of colorant were studied by using an NMR spectrometer (AVANCE III HD 500, Bruker, Bremen, Germany). SEM (TESCAN MIRA LMS, Kohoutovice, Czech Republic) and transmission electron microscopy (TEM) were used to investigate the morphological properties and composition of BBC and BBC-HAP (TEM, JEOL JEM 2100F). In the microscope, an energy dispersive X-ray spectrometer was added (EDX; SMART, EDAX Inc., Mahwah, NJ, USA). The zeta potential was determined using a Zeta potential analyzer (Nano-ZS90X, Malvern, UK). The chemical functional groups of the materials were determined using Fourier transform infrared spectroscopy (FTIR; IRTracer-100, Shimadzu, Japan). The thermal stability of bagasse before and after activation was investigated using thermogravimetric analysis (PerkinElmer TGA 4000, Waltham, MA, USA). An X-ray diffractometer was used to determine the X-ray diffraction (XRD) pattern (Rigaku Ultima IV, Tokyo, Japan). The elemental composition and surface chemical states of BBC-HAP and BBC-HAP-Colorant surfaces were analyzed with the X-ray photoelectron spectroscopy (XPS) technique (K-Alpha, Thermo Fisher Scientific, Waltham, MA, USA).

### 2.1. Preparation of Bagasse Biochar (BBC)

Bagasse was cleaned completely with tap water, then moistened with distilled water and dried overnight at 60 °C. The dried bagasse was crushed and screened to 60 mesh size before being kept in a dry vacuum atmosphere. The dried bagasse powder (6 g) was soaked in 35% phosphoric acid at a 1:5 (*w/v*) ratio, mixed uniformly, and baked for 12 h at 105 °C. Under continuous nitrogen flow, activated bagasse was heated at 450 °C for 2.5 h [20]. The solid biomass was cooled to room temperature, rinsed with 0.1 M sodium hydroxide solution, washed with distilled water to a neutral pH value, dried for 24 h at 60 °C, crushed and screened to 100 mesh size, and finally stored in a dryer.

### 2.2. Synthesis of Bagasse Biochar Coated Hydroxyapatite (BBC-HAP) Composite

Solution A (40 mL) containing 0.47 g Ca(NO_3_)_2_·4H_2_O and Solution B (30 mL) containing 0.16 g (NH_4_)_2_HPO_4_ were prepared in distilled water. Next, 1.5 g of BBC was slowly added to solution A and mixed thoroughly at 450 rpm with magnetic stirring for 1 h. Solution B was continued to be slowly added to the solution A mixture using a 1 mL syringe under magnetic stirring and the pH of the solution was maintained at 10.0–10.5 with ammonia solution. After rapid stirring for 2 h, the solution was left to age at room temperature for 24 h [21]. The BBC-HAP precipitate was extracted using a vacuum filtration pump, then washed several times with distilled water and dried at 60 °C for 12 h. The BBC-HAP material was crushed into powder, sieved using a 100 mesh sieve and placed in sample bottles before being used for adsorption studies. HAP was produced under the same experimental conditions as a control.

### 2.3. Extraction of Colorant from Sugarcane Juice

Sugarcane juice was filtered through a ceramic membrane to remove macromolecular impurities inside. Ten g macroporous adsorption resin was soaked in 50 mL anhydrous ethanol for 24 h (information related to the macroporous adsorption resin used is presented in Supplementary Table S1), then vacuum filtrated, and anhydrous ethanol was repeated used until the filtrate was no longer white and turbid. Distilled water was then used to wash the resin to remove ethanol. The colorant was extracted from 10 g of treated resin in an ion exchange column against 250 mL of membrane clear juice. After the adsorption was completed, the column was rinsed with distilled water. Anhydrous ethanol solution (100 mL) was used to elute the colorant. The color extract was concentrated in a rotary evaporator, and then freeze-dried for 36 h to obtain a dry colorant. The dried colorant was dissolved in water to obtain the colorant solutions (different concentrations) for the adsorption experiments.

### 2.4. Batch Adsorption Experiments

In a conical flask holding 20 mL of colorant solution, colorant adsorption by BBC-HAP was done in batches. The effects of major adsorption process factors on colorant removal were examined, including adsorbing materials (HAP; BBC; HAP-BBC), adsorbent doses (0.25–1.25 mg/mL), pH values (3–11), contact time (0–240 min), starting colorant concentrations (150–250 mg/L), sucrose concentrations (0–20 °Brix), and potassium ionic strength (0–2000 mg/L). We also compared the adsorption performance of BBC-HAP on colorants with that of commercial activated carbon (Hongsheng, Macklin, Sinopharm, and Guanghua Group), ion exchange resins (A722, D201, and D301), and fiber. Since the colorant is relatively active, the temperature increase will affect its initial color value, but it is stable at a temperature of 300 K. Therefore, all adsorption experiments were performed in a shaking incubator at 300 K with a shaking speed of 130 rpm. The change in concentration of the colorant before and after adsorption by BBC-HAP was measured with a spectrophotometer at 420 nm (SP-752, Spectrum, Shanghai, China). All solutions were filtered through a 0.45 μm filter membrane to remove any suspended impurities that might affect the analytical results before measuring absorbance. The concentration and absorbance values before and after adsorption were used to calculate the extent of residual colorant remaining in the solution. The following equations were used to determine the adsorption capacity at time t and the colorant removal rate of BBC-HAP [22]:

(1) $$q_{t}=\frac{\left(C_{0}-C_{e}\right)\cdot V}{m}$$

(2) $$q_{e}=\frac{\left(C_{0}-C_{e}\right)\cdot V}{m}$$

(3) $$R=\frac{\left(C_{0}-C_{t}\right)}{C_{0}}\times 100\%$$

where *q_t_* (mg/g) and *q_e_* (mg/g) are the adsorption capacities of BBC-HAP for colorant at time *t* and equilibrium, respectively; *C*_0_ (mg/L), *C_t_* (mg/L), and *C_e_* (mg/L) denote the concentrations of colorant initially, at time *t*, and at equilibrium, respectively; *V* (L) is the volume of colorant solution; *m* (g) is the adsorbent dosage; *R* (%) is the removal rate of colorant by BBC-HAP.

All experiments were performed in triplicate, and the data were presented as mean ± standard deviation. Statistical analyses were conducted with SPSS software (version 20.0). One-way analysis of variance was performed using Tukey’s honest significant difference post hoc test to detect significant differences among means. The means were considered significantly different at the 5% (*p* < 0.05) level.

## 3. Results and Discussion

### 3.1. Characterization of Colorant

#### 3.1.1. NMR Analysis

**Figure 1:**
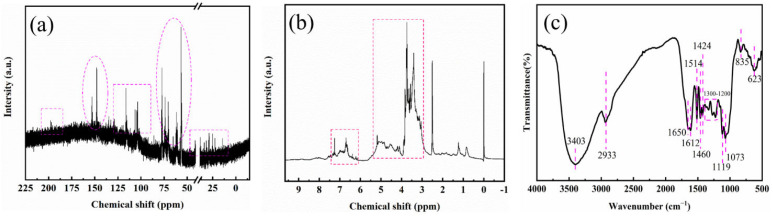
Chemical characterization of the colorant by: (**a**) ^13^C-NMR spectra (**b**) ^1^H-NMR spectra (**c**) FTIR spectra.

The ^13^C-NMR and ^1^H-NMR spectra of the colorants were measured by NMR spectroscopy using DMSO as solvent. Figure 1a shows the ^13^C-NMR spectrum of colorant, with peaks between 0 ppm and 50 ppm, indicating the presence of saturated alkanes in the colorant. The characteristic peaks of the alcoholic hydroxyl groups were 60 ppm to 80 ppm, the absorption peak of amino carbon connected to glucose was around 61.5 ppm, and the non-aromatic carbon of gallate related components also had absorption peaks in the range of 67.77~76.33 ppm [23]. It has been reported that the α-C absorption peak of amines is between 20 and 75 ppm [24,25]. The broad peak region from 90 ppm to 130 ppm was caused by the chromophore unsaturated C=C bonds of the colorant. The characteristic peaks from 147 ppm to 153 ppm may be caused by carboxyl/carboxylate drift. In addition, the peak at 200 ppm was attributed to ketones [26,27]. According to Figure 1b, in the ^1^H-NMR spectrum of colorant, the peaks at 2.5 and 3.5 ppm were characteristic of the solvents DMSO and H_2_O, respectively. The broad peak region at 2.83 to 5.13 ppm was mainly due to the Maillard reaction of glucose with phenolic amino acids [24,25], and the characteristic proton region signal of the melanoidin fraction was approximately 3.0 to 4.6 ppm. At 6.10 to 7.16 ppm, it was mainly the absorption peak region of phenolic/aromatic compounds [28]. In summary, the substances in the colorant composition mainly include two types of substances; one is the Maillard product of reducing sugars and amino acids, which is the main component of the colorant in sugarcane juice. The other category is aromatic compounds of phenolic esters, which are mainly phenolic compounds, and the content of aromatic compounds (i.e., phenolic substances) is low because the sugarcane juice production process is at high temperature for a long time, and phenolic substances easily transformed and degraded [29,30].

#### 3.1.2. FTIR Analysis of Colorant

The colorant was further characterized by FTIR, and the functional groups were determined to elucidate the structure of colorants. In Figure 1c, the peak at 3403 cm^−1^ is attributed to the stretching vibration of the –OH groups [31]. The peak at 2933 cm^−1^ is caused by the C–H bond from methylene and/or methyl, which originates from the catenated carbon-containing materials in the colorant [32]. The bands at 1650 and 1612 cm^−1^ correspond to the characteristic peak of C=C and C=O bonds [33]. In addition, the absorption peak between 1300–1200 cm^−1^ is the C-O stretching vibration absorption peak of phenols [34]. The peak at 1073 cm^−1^ is attributed to the C–O bond in colorant [33,34]. The bands at 1424 and 835 cm^−1^ are ascribed to C–H and/or –CH_3_ deformation [26,33,34]. The peaks at 1460 and 623 cm^−1^ most probably result from O–H bonds in the colorants [26,34]. On the basis of the FTIR data the colorant extract is likely to contain flavonoids, phenolics and 5 hydroxymethyl furfural.

### 3.2. Characterization of BBC-HAP

#### 3.2.1. SEM-EDX and TEM Analysis

**Figure 2:**
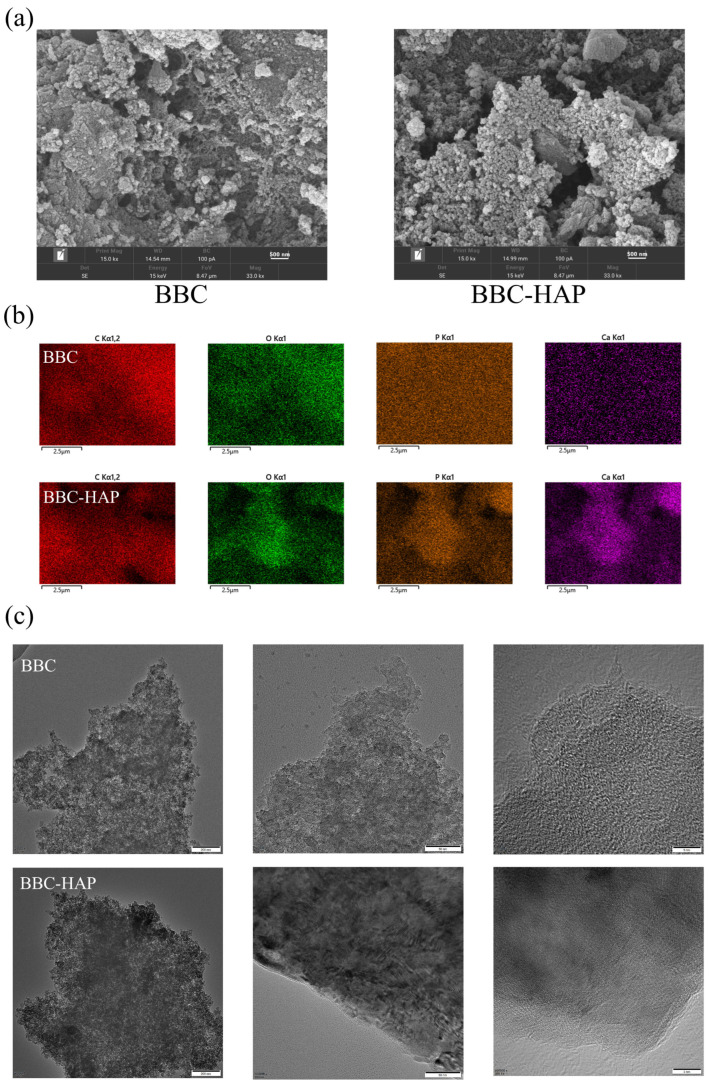
Study of the morphological properties of the BBC and BBC-HAP adsorbents by: (**a**) SEM micrographs (**b**) C, O, P, and Ca EDX elemental mapping images and (**c**) TEM images magnified at 200 kv.

Figure 2a shows the SEM micrographs of the BBC and BBC-HAP respectively. Irregularly shaped rough surfaces and abundant porous structures were observed. The irregular surfaces and porous structures contribute to improve adsorption capacity. The white nanocrystals in the SEM image of BBC-HAP are surface-synthesized HAP coatings. The EDX spectrum shows the elemental distribution of C, O, Ca, and P (Figure 2b). As expected, the Ca content of BBC-HAP increased significantly (Table 1), indicating that the chemical structural composition of BBC-HAP has changed significantly. The surface analysis of the EDX results is consistent with the results of SEM micrographs, showing that HAP crystals are formed on the surface. The size, shape, and microstructures of BBC and BBC-HAP were revealed by TEM images (Figure 2c), indicating the presence of a unique, layered porous structure with bulky multi-layered aggregations, which was similar to the SEM analysis.

#### 3.2.2. N_2_ Adsorption–Desorption Experiments

**Figure 3:**
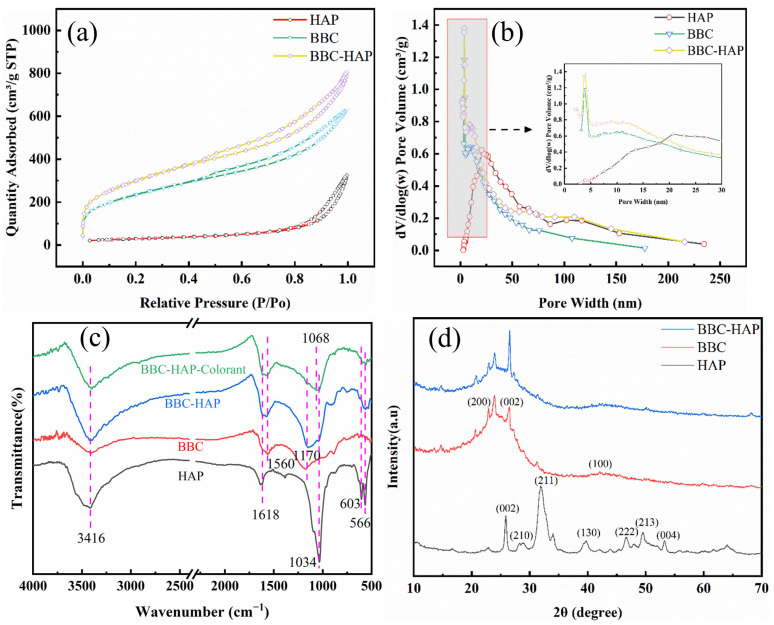
(**a**) N_2_ adsorption–desorption isotherms and (**b**) pore size distributions of HAP, BBC and BBC-HAP. (**c**) FTIR spectra of HAP, BBC, BBC-HAP and BBC-HAP-Colorant. (**d**) XRD patterns of HAP, BBC and BBC-HAP.

Figure 3a shows that the isotherms of HAP, BBC and BBC-HAP present typical type IV curves (P/P_0_ > 0.7). The desorption curve is inconsistent with the adsorption curve, showing an obvious hysteresis loop, indicating that the interaction between the adsorbent and the adsorbate is strong, and the adsorption amount in the extremely low-pressure range (P/P_0_ < 0.01) is not equal to zero. Figure 3b shows that BBC and BBC-HAP have mesoporous structures, and the average pore diameter of BBC-HAP is 4.56 nm, which is smaller than that of BBC (4.63 nm) (Table 2). The Brunauer–Emmett–Teller (BET) specific surface area of the BBC is 832.55 m^2^/g, which is lower than that of the BBC-HAP (1067.67 m^2^/g). BBC-HAP has a larger specific surface area which can provide more active binding sites to capture colorants [35,36,37].

#### 3.2.3. FTIR Analysis of HAP, BBC, BBC-HAP and BBC-HAP-Colorant

The FTIR spectral profiles recorded for HAP, BBC, BBC-HAP and BBC-HAP-Colorant are shown in Figure 3c. The hydroxyl group in BBC or HAP is represented by the stretching vibration of the -OH group at 3416 cm^−1^ [38,39]. The band located at 1618 cm^−1^ was assigned to the −COO in CO_3_^2−^ in HAP [40], and the 1560 cm^−1^ and 1170 cm^−1^ peaks observed in the spectrum profile for BBC are attributed to the stretching vibration of the C-C bond and C-O, respectively [41,42,43]. In addition, the HAP contains a P-O (V_3_) band of 1034 cm^−1^ and O-P-O (V_4_) bands of 603 cm^−1^ and 566 cm^−1^ [35]. The spectral profiles recorded for BBC-HAP and BBC-HAP-Colorant have a weakened peak at 1170 cm^−1^, indicating that C-O is involved in the adsorption of the colorant. A new peak appeared in 1068 cm^−1^, a shift to lower wavenumber by 5 cm^−1^, from characteristic colorant peak indicates successfully molecular interactions between the colorant and BBC-HAP, perhaps through intermolecular H-bonding or similar interactions.

#### 3.2.4. XRD Analysis

The XRD patterns are shown in Figure 3d. In terms of peak position and relative intensity, the recorded XRD patterns matched the hydroxyapatite (HAP) reference well. The (002), (210), (211), (130), (222), (213) and (004) reflection peaks of hydroxyapatite were measured at 2θ = 25.88°, 28.68°, 32.04°, 39.62°, 46.64°, 49.52°, and 53.19°, respectively [44]. The diffraction pattern of the BBC shows a crystalline phase and an amorphous phase, attributed to the presence of cellulose and hemicellulose in the BBC precursor. Polyethyleneimine-modified coffee waste has been reported to contain cellulose with similar amorphous and crystalline phases [45]. The spectra represent the (200), (002), and (100) planes at 2θ = 20.9°, 26.5°, and 42.5°, respectively (JCPDS Card No. 41-1487). The peak of BBC-HAP is enhanced at 2θ = 26.5°, which may be the result of the superposition of HAP on BBC for the composite material. A similar result has been discussed for the activated carbon composite hydroxyapatite [19].

#### 3.2.5. XPS Analysis of BBC-HAP and BBC-HAP-Colorant

**Figure 4:**
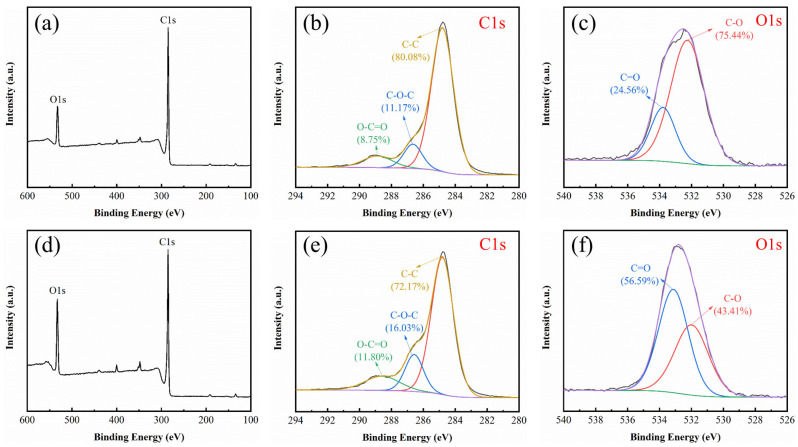
Study of elemental composition and surface chemical states of BBC-HAP and BBC-HAP-Colorant by (**a**) and (**d**) XPS spectra, (**b**) and (**e**) C1s spectra and (**c**) and (**f**) O1s spectra.

The elemental composition and surface chemical states of BBC-HAP and BBC-HAP-Colorant were analyzed by XPS, and the results are shown in Figure 4. The major elements of BBC-HAP and BBC-HAP-Colorant were C (87.01% vs. 81.53%) and O (12.99% vs. 18.47%). Compared to BBC-HAP, the elemental content of O in BBC-HAP-Colorant was increased due to the large amount of oxygen-containing functional groups in the colorant, which indirectly indicates the successful adsorption of the colorant in BBC-HAP. In order to further determine the surface functional group structures of BBC-HAP and BBC-HAP-Colorant, separate peak fits were performed. The results of the C1s fits are shown in Figure 4b,e. 288.86/288.57, 286.65/286.57, 284.80/284.80 indicate O-C=O, C-O-C, and C-C, respectively [46], in which the content of C-C slightly decreases and the content of O-C=O and C-O-C slightly increases. The results of O 1 s fitting are shown in Figure 4c,f. 522.79/533.12 and 532.26/531.99 indicate C = O and C-O, respectively [35], and it can be found that C = O is shifted. It is important that the content of C = O is significantly enhanced while the content of C-O is decreased obviously, which indicates that the oxygen-containing functional groups on the surface of BBC-HAP are involved in the adsorption of colorants.

### 3.3. Adsorption Experiments

#### 3.3.1. Effect of Adsorbing Materials

**Figure 5:**
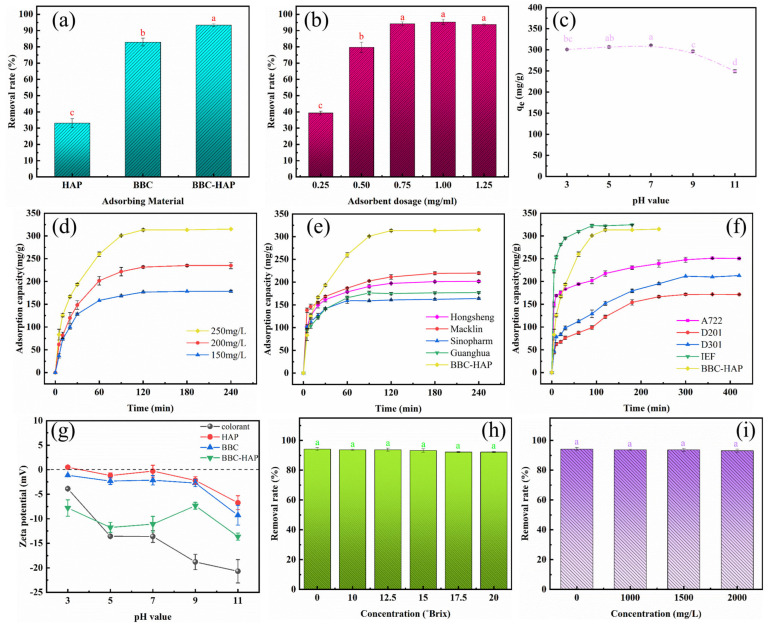
Adsorption experiments (General conditions: dosage, 0.75 mg/mL; temperature, 300 K; pH, 7; time, 6 h and initial colorant concentration, 250 mg/L). (**a**) Variations in colorant removal by different adsorbents. (**b**) Effect of BBC-HAP dosage on colorant removal. (**c**) Effect of pH on the equilibrium adsorption capacity of BBC-HAP for colorant. (**d**) Effects of initial colorant concentration and contact time on the adsorption capacity of BBC-HAP. (**e**) Colorant adsorption by four activated carbons. (**f**) Colorant adsorption by three commercial resins and one ion exchange fiber. (**g**) Zeta potential of colorant, HAP, BBC, and BBC-HAP. (**h**) Effect of sucrose solution concentration on the adsorption capacity of BBC-HAP for colorant (time, 2 h). (**i**) Effect of KCl concentration on the adsorption capacity of BBC-HAP for colorant (time, 2 h). Data represent mean values ± SD (*n* = 3). Different letters above the error bars indicate significant differences according to the Tukey’s HSD test (*p* < 0.05).

Figure 5a shows the colorant removal efficiencies of HAP, BBC, and BBC-HAP. Under the same parameters, the colorant removal efficiency of BBC (82.94%) was found to be significantly better than the colorant removal efficiency of HAP (33.12%). The dense porous structure and large surface area of BBC account for the improved effectiveness. The improvement in efficiency can be attributed to the increased surface area of the composite by HAP and the involvement of HAP in co-adsorption. It shows that the composite of BBC and HAP is an effective measure when applied to the decolorization of cane juice.

#### 3.3.2. Effect of BBC-HAP Dosage

The effects of adsorbent dose on colorant removal by BBC-HAP are shown in Figure 5b. When the dose of BBC-HAP was increased from 0.25 to 0.75 mg/mL, the colorant removal rate increased from 39.31% to 94.25% because the adsorbent dose directly increased the active adsorption sites. However, when the BBC-HAP dose exceeded 0.75 mg/mL, the increase in colorant removal was negligible. Importantly, reducing the dosing of BBC-HAP will reduce the waste of resources and lower the operating costs of the sugar process. 0.75 mg/mL of BBC-HAP was determined as the optimal BBC-HAP dose from an economic standpoint and for industrial uses.

#### 3.3.3. Effect of pH

Since the physicochemical properties of the colorant and BBC-HAP in solution are closely related to pH, pH has a great influence on the adsorption capacity of BBC-HAP on the colorant. Figure 5c investigates the effect of the initial solution pH values between 3 and 11 on the ability of BBC-HAP to adsorb colorants. In the range of pH 3 to 7, the adsorption capacity slightly increases, perhaps because of increased interaction of the carbon functional groups and the electropositive HAP surface. The adsorption is highest at pH 7. In the range of pH 7 to 11, the adsorption effect is greatly reduced. This is because the physical and chemical properties of the colorant have changed in an alkaline environment, not only because of ionizing anions, but also because of the increasing initial color value. The zeta potentials of colorant and adsorbent materials at different pH levels are shown in Figure 5g. The adsorption material and colorant are basically negatively charged in the range of pH 3–11, indicating that the adsorption material does not rely on the mutual attraction between positive and negative charges, but relies on the physical nature of the pores and the chemical functional groups as shown by the FTIR data. The zeta potential of the colorant and the adsorbents decrease with increasing pH, and hence repulsion occurs between them, thereby hindering effective adsorption.

#### 3.3.4. Effects of Contact Time and Initial Colorant Concentration

**Figure 6:**
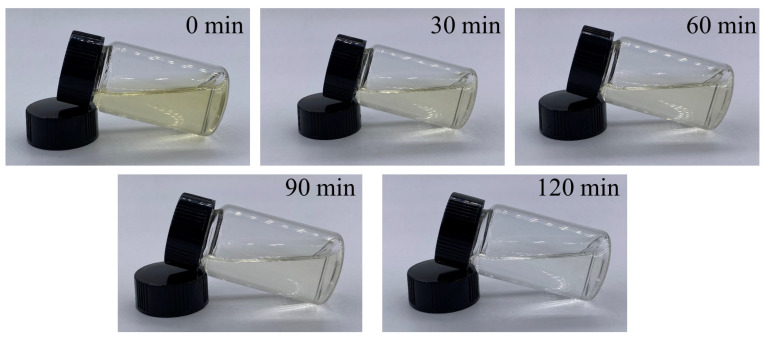
Photographs of colorant solutions before and after adsorption for 30–120 min by BBC-HAP (dosage, 0.75 mg/mL; temperature, 300 K; pH, 7; and initial colorant concentration, 250 mg/L).

As shown in Figure 5d, the adsorption capacity of BBC-HAP on the colorant increased rapidly in the first 30 min, which may be due to the high affinity between the colorant and the BBC-HAP surface during this period. With the increase of the adsorption time of colorant, the adsorption sites of BBC-HAP were gradually occupied and the adsorption rate became slower and slower, and finally the adsorption saturation reached the adsorption equilibrium. At different initial colorant concentrations, all adsorption experiments reached equilibrium at about 120 min. Figure 6 shows the pictures of 250 mg/L colorant solution before being adsorbed by BBC-HAP at 5, 30, 60 and 120 min. From the light brown before adsorption, the color gradually becomes lighter until it is almost transparent and colorless. The initial colorant concentration was closely connected to the colorant adsorption by BBC-HAP. When the initial colorant concentration was increased from 150 to 250 mg/L, the adsorption of colorant at 120 min increased from 176.67 to 313.33 mg/g. This phenomenon indicates that at higher colorant concentrations, the contact possibility between the adsorbent and the colorant adsorbate of BBC-HAP increases, and at higher concentrations the colorant can also be dispersed into BBC-HAP faster.

#### 3.3.5. Comparison of Performance with Activated Carbon, Commercial Resin and Ion Exchange Fiber

Figure 5e,f show the adsorption capacities of four commercially available activated carbons (Hongsheng, Macklin, Sinopharm, and Guanghua Group), three commercially available ion exchange resins (A722, D201, and D301), and one ion exchange fiber for colorants, respectively. The information related to the activated carbon, ion exchange resin and fiber used are listed in Supplementary Tables S2 and S3, respectively. Figure 5e shows the variation of the adsorption capacity of the four commercially available activated carbon samples obtained from different manufacturers on the colorants. When the adsorbent dosage was 0.75 mg/mL, the pH value was 7, the temperature was 300 K, and the initial colorant concentration was 250 mg/L, the adsorption capacities of the four commercial activated carbons ranged from 162.17 mg/g to 219.83 mg/g when adsorption reached equilibrium, which was lower than that of BBC-HAP (313.33 mg/g). The adsorption capacities of the four commercial activated carbons at equilibrium ranged from 162.17 mg/g to 219.83 mg/g. As shown in Figure 5f for the resins A722, D201, D301 and IEF, they reached kinetic equilibrium at 240, 300, 300 and 90 min with adsorption amounts of 250.50, 171.67, 211.67 and 322.17 mg/g, respectively. This result showed that under the same conditions, the adsorption rate of BBC-HAP for this colorant was significantly higher than that of the three commercial resins, and slightly lower than that of IEF. The commercial price of IEF is much higher than the other adsorbents. Therefore, BBC-HAP not only has good adsorption performance but also has a price advantage, and its application to decolorization of cane juice has good potential.

#### 3.3.6. Effect of Sucrose Solution and Potassium Ion Concentration

The most prevalent organic and inorganic components in sugarcane juice are sucrose and potassium ions. The content of sugarcane juice sucrose (°Brix) is usually between 10 and 20 °Brix. We evaluated the effects of sucrose concentration and potassium ion strength on BBC-HAP adsorption performance to further study the viability of BBC-HAP for the decolorization of sugarcane juice, and the findings are given in Figure 5h,i. The adsorption capacity of BBC-HAP for colorant stays relatively consistent when the concentration of sucrose solution is 0–20 °Brix and the concentration of potassium ions is 0–2000 mg/L, with only minor changes in the samples. As a result, BBC-HAP can be used to decolorize sugarcane juice.

#### 3.3.7. Desorption and Regeneration

**Figure 7:**
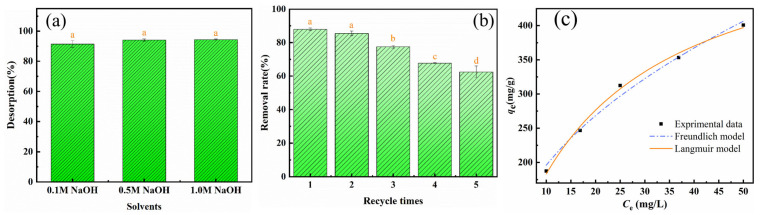
Desorption, regeneration and adsorption isotherms of BBC-HAP (adsorbent dosage, 0.75 mg/mL; pH, 7; temperature, 300 K; and initial concentration, 250 mg/L). (**a**) Regeneration with different analytical agents. (**b**) Effects of the number of regeneration cycles on colorant removal and (**c**) Langmuir and Freundlich model descriptions of colorant adsorption onto BBC-HAP (initial concentration between 150–350 mg/L). Data represent mean values ± SD (*n* = 3). Different letters above the error bars indicate significant differences according to the Tukey’s HSD test (*p* < 0.05).

To improve the utilization cyclicality and sustainability of BBC-HAP, 0.1 M NaOH, 0.5 M NaOH, and 1.0 M NaOH were tested as regenerants (Figure 7a). The use of 0.5 M NaOH gave the highest removal of 94.39% and was selected as the regenerant, and its cyclic adsorption performance was investigated (Figure 7b). The figure shows that after five adsorption-desorption (regeneration) cycles, the adsorption capacity of BBC-HAP for colorant removal remained above 62%. Meanwhile, we found that the color of NaOH solution gradually changed from colorless to yellow during the resolution process, which indicated that the colorant molecules adsorbed by BBC-HAP were gradually separated from BBC-HAP and dissolved in NaOH solution during the resolution process. The high adsorption capacity and good recyclability of the BBC-HAP composite make it an effective adsorbent for colorant removal from sugar juice, which is clearly of practical importance for future industrial applications.

### 3.4. Adsorption Isotherms

The adsorption isotherm plays a non-negligible role in the adsorption process, which reveals the connection between the equilibrium adsorption capacity of the adsorbent material and the adsorption equilibrium concentration under isothermal conditions. Adsorption isotherms can help one understand how adsorption systems behave [22,47].

The Langmuir isotherm model is used to simulate the homogeneous monolayer adsorption behavior defined by Equation (4) [48]:

(4) $$q_{e}=\frac{q_{m}\cdot k_{L}\cdot C_{e}}{1+k_{L}\cdot C_{e}}$$

where *q_m_* (mg/g) is the maximum adsorption capacity of BBC-HAP for colorant and *k_L_* (L/mg) is the Langmuir adsorption constant. The heterogeneous multilayer adsorption behavior is described by the Freundlich isotherm model, which is represented by Equation (5) [49]:

(5) $$q_{e}=k_{F}\cdot C_{e}^{\frac{1}{n}}$$

where *n* is a parameter used to quantify the intensity of the interaction between BBC-HAP and colorant molecules, and *k_F_* (mg/g) is the Freundlich adsorption constant. The statistical parameter *Adj.R*^2^ is generated using Equation (6) to assess the fitting outcomes of the isotherm models to the experimental data.

(6) $$Adj.R^{2}=1-\frac{\sum {\left(q_{exp}-q_{cal}\right)}^{2}}{\sum {\left(q_{exp}-q_{mean}\right)}^{2}}\cdot \frac{N-1}{N-P}$$

where *q_exp_* (mg/g) and *q_cal_* (mg/g) are the experimental and model-calculated adsorption capacities, respectively; *q_mean_* (mg/g) is the average experimental adsorption capacity; *N* is the number of experimental data; *P* is the number of model parameters.

The adsorption isotherm experiments were carried out at a temperature of 300 K. The adsorption of 150–350 mg/L colorant by BBC-HAP was investigated at a dosing rate of 0.75 mg/g. The results of fitting the Langmuir and Freundlich models to the experimental data are shown in Figure 7c, and the corresponding parameters are listed in Table 3. The fitting results show that the Langmuir model has a higher *Adj.R*^2^ than the Freundlich model, indicating that the adsorption process of BBC-HAP on the colorant is more consistent with the Langmuir model, which indicates that the BBC-HAP composite has a uniform adsorption surface and the adsorption of colorant on BBC-HAP is a monolayer adsorption process [50].

### 3.5. Adsorption Kinetics

In order to better utilize the BBC-HAP adsorption process for the separation of sugarcane juice colorants, the reaction kinetics were investigated. The pseudo-first-order kinetic model (PFO) is the most commonly used model to determine whether the adsorption process is physisorption, while the pseudo-second-order kinetic model (PSO) is the most commonly used model to determine whether the adsorption process is chemisorption-dominated, and the adsorption process is determined by comparing the fitted correlation coefficients (*Adj.R*^2^) of the model and experimental adsorption data to determine whether the adsorption process is a physisorption process or a chemisorption process. However, in the actual adsorption process, the result is not a single one, but, on the contrary, it is often complex, as it may include both physical and chemical adsorption. Therefore, we propose a hybrid kinetic model combining a pseudo-first-order kinetic model and a pseudo-second-order kinetic model (PFO-PSO), which is solved by the Runge-Kutta method in matlab software to determine the kinetic behavior of colorant adsorption on BBC-HAP. The linearized forms of the PFO, PSO and PFO-PSO models are expressed as follows [51]:

(7) $$\ln\left(q_{e}-q_{t}\right)=lnq_{e}-k_{1}t$$

(8) $$\frac{t}{q_{t}}=\frac{1}{k_{2}q_{e}^{2}}+\frac{t}{q_{e}}$$

(9) $$\frac{dq_{t}}{dt}=k_{PFO}\left(q_{m}-q_{t}\right)+k_{PSO}{\left(q_{m}-q_{t}\right)}^{2}$$

where *k_1_* (1/min) is the PFO rate constant, *k_2_* [g/(mg⋅min)] is the PSO rate constant and *k_PFO_* (1/min), *k_PSO_* [g/(mg⋅min)] are the PFO and PSO rate constants of the PFO–PSO combined model, respectively.

**Figure 8:**
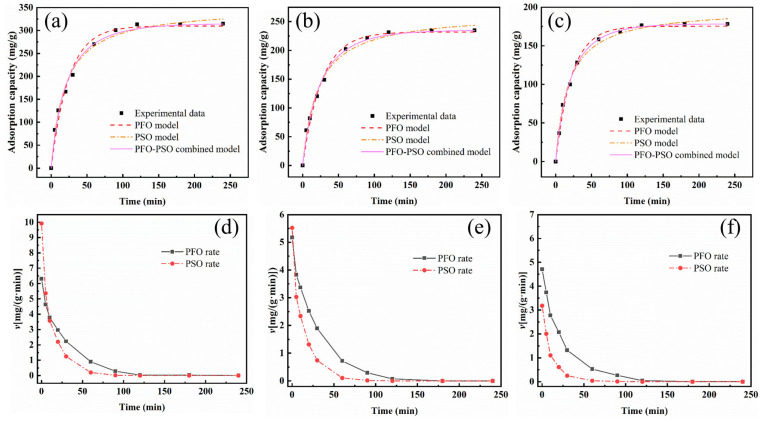
Experimental data of colorant adsorption onto BBC-HAP at initial concentrations of (**a**) 250, (**b**) 200, and (**c**) 150 mg/L predicted by various kinetic models. Contributions of PFO and PSO rates over the whole process of BBC-HAP adsorption of colorant at the initial concentrations of (**d**) 250, (**e**) 200, and (**f**) 150 mg/L (adsorbent dosage, 0.75 mg/mL; pH, 7; temperature, 300 K).

Figure 8a–c show the results of the PFO, PSO, and combined PFO-PSO models fitted to BBC-HAP for different initial colorant concentrations (250, 200, and 150 mg/L). The parameter values of the fitted results are summarized in Table 4. According to Figure 8a–c and the values of *Adj.R*^2^ (Table 4), we can see that the *Adj.R*^2^ of the combined PFO-PSO model is higher than that of the PFO and PSO models alone, which indicates that the adsorption process of BBC-HAP on colorants is a physicochemical co-adsorption process, which may also account for the higher adsorption effect than that of commercial adsorbents. Interestingly, the *Adj.R*^2^ of the PSO model is higher than that of the PFO model, which indicates that chemisorption is dominant and physical adsorption is supplementary in the adsorption of colorants by BBC-HAP. To further verify the dominant chemical and physical mechanisms, the PFO and PSO rates were estimated for the combined PFO-PSO model using Equations (10) and (11) [52]:

(10) $$v_{PFO}=k_{PFO}\left(q_{m}-q_{t}\right)$$

(11) $$v_{PSO}=k_{PSO}{\left(q_{m}-q_{t}\right)}^{2}$$

where *v_PFO_* [mg/(g⋅min)] and *v_PSO_* [mg/(g⋅min)] are the PFO and PSO rates of the PFO–PSO combined model, respectively.

Figure 8d–f show the variation of the adsorption rates versus time for PFO and PSO at initial colorant concentrations of 250, 200 and 150 mg/L. The trend of variation is comparable at different starting colorant concentrations, with the adsorption rate being maximum at the beginning and then decreasing with increasing time until adsorption equilibrium is reached. In the adsorption process of BBC on the colorant, the higher the concentration, the higher the rate of chemisorption, and conversely, at low concentrations, the rate of physical adsorption is higher than the rate of chemisorption. This indicates that at high concentrations, the colorant can quickly bind to the adsorption sites in BBC-HAP through oxygen-containing functional groups, while at low concentrations the colorant can only enter BBC-HAP by intraparticle diffusion. Overall, the new combined PFO-PSO model can clearly reflect the dynamic process of chemisorption and physisorption in the whole adsorption process [27]. It offers new perspectives on adsorption kinetic dynamics and makes some theoretical and practical advances.

### 3.6. Mass Transfer Mechanisms

**Figure 9:**
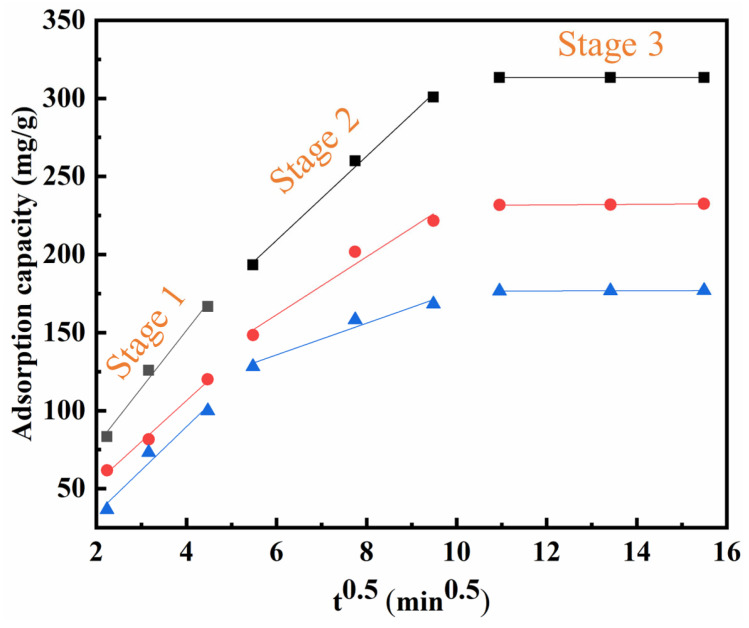
Fitting of intraparticle diffusion model for colorant adsorption by BBC-HAP.

The mass transfer mechanism of the BBC-HAP adsorbed colorant is studied in Figure 9. Adsorption is a process in which the adsorbent is gradually transferred from the liquid to the solid. To investigate the diffusion mechanism in the adsorption of colorants, Weber and Morris developed a linear intraparticle diffusion (IPD) model Equation (12) to represent the mass transfer process [53].

(12) $$q_{t}=K_{IPD}t^{0.5}+c$$

where *q_t_* (mg/g) is the adsorption capacity of BBC-HAP on the colorant at time *t* (min); *K_IPD_* (mg/g/min^0.5^) is the IPD rate constant, and *c* (mg/g) is the intercept point of the linear plot of qt versus *t*^0.5^.

The *Adj.R*^2^ fitted by the IPD model were all higher than 0.9, indicating that intraparticle diffusion drives the adsorption of the colorant on BBC-HAP to some extent. As shown in Figure 9, the adsorption of colorant on BBC-HAP involves many processes, and the figure shows a multilevel linear relationship from the origin [54]. The first stage is mainly external surface adsorption (pore adsorption), where the colorant reaches the external surface of the adsorbent from the native solution across the liquid film. The second linear stage is the intraparticle diffusion, dominated by chemisorption, where the colorant diffuses through the interior of the BBC-HAP and finally reaches the adsorption site. The third stage is the final equilibrium stage, dominated by physical adsorption, where the colorant slowly moves from the larger pores into the micropores by pore diffusion. During the adsorption of colorant by BBC-HAP, the rate constants are arranged as follows: k_1_-int > k_2_-int > k_3_-int (Table 5). This proves that it is influenced by external mass transfer, chemisorption and physical adsorption throughout the adsorption of colorant by BBC-HAP, which is consistent with the fitted results of the combined PFO-PSO model.

## 4. Conclusions

In summary, we have reported a novel porous BBC-HAP adsorbent using waste biomass bagasse as precursor via a combination of one-step carbonization process with HAP. The porous carbon surface containing HAP nanoparticles shows excellent adsorption capacity for colorants in sugarcane juice, up to 313.33 mg/g (BBC-HAP dosage, 0.75 mg/mL; Initial concentration, 250 mg/L; temperature, 300 K; and pH, 7). By introducing other nanoparticles and expanding the surface area of materials, the adsorption behavior between colorant and carbonaceous materials can be improved, which is expected to improve the adsorption performance of other carbon-based sorbents.

## Figures and Tables

**Table 1:** Weight percentages of various elements present in BBC and BBC-HAP.

Element	C (%)	O (%)	P (%)	Ca (%)
BBC	77.95 ± 0.15	16.24 ± 0.15	5.81 ± 0.06	0.00 ± 0.03
BBC-HAP	55.08 ± 0.23	21.05 ± 0.23	4.67 ± 0.11	19.21 ± 0.13

Data represent mean values ± SD (*n* = 3).

**Table 2:** HAP, BBC, and BBC-HAP surface areas, total pore volumes, and average pore diameters.

Sample	Surface Area (m^2^/g)	Total Pore Volume (cm^2^/g)	Average Pore Size (nm)
HAP	103.81	0.48	18.58
BBC	832.55	0.96	4.63
BBC-HAP	1067.67	1.22	4.56

**Table 3:** Fitting parameters of the Langmuir and Freundlich models to the experimental data of colorant adsorption onto BBC-HAP.

Model	Parameters	*Adj.R* ^2^
Langmuir model	q_m_ = 560.41 ± 19.72 mg/g; k_L_ = 0.05 L/mg	0.993
Freundlich model	k_F_ = 68.99 ± 7.58 mg/g; n = 2.21 ± 0.03	0.984

Data represent mean values ± SD (*n* = 3).

**Table 4:** Kinetic parameters of colorant adsorption by BBC-HAP (adsorbent dosage, 0.75 mg/mL; pH, 7; and temperature, 300 K).

Concentration (mg/L)		150	200	250
*q_e_*, exp (mg/g)		178.33 ± 1.18	240.00 ± 7.07	313.33 ± 2.36
PFO	*q_e_*_,cal_ (mg/g)	178.06	243.94	309.75
	*k*_1_ (1/min)	0.04	0.042	0.041
	*Adj.R* ^2^	0.994	0.984	0.983
PSO	*q_e,_*_cal_ (mg/g)	203.44	276.84	351.14
	*k*_2_ [g/(mg·min)]	0.00024	0.00019	0.00015
	*Adj.R* ^2^	0.996	0.993	0.992
PFO-PSO	*k*_PFO_ (1/min)	0.0264	0.022	0.02
	*k*_PSO_ [g/(mg·min)]	0.0001	0.0001	0.0001
	*Adj.R* ^2^	0.998	0.995	0.993

Data represent mean values ± SD (*n* = 3).

**Table 5:** The intra-particle diffusion parameters for colorant adsorption by BBC-HAP.

Concentration mg/L	k_1_-Int	*Adj.R* ^2^	k_2_-Int	*Adj.R* ^2^	k_3_-Int	*Adj.R* ^2^
250	36.8633	0.976	26.9377	0.992	0.0044	0.995
200	26.2982	0.987	18.5470	0.935	0.1709	0.970
150	27.7951	0.929	10.1364	0.917	0.0635	0.956

## Data Availability

Data is contained within the article or Supplementary Material.

## Supplementary Materials

The following supporting information can be downloaded at: https://www.mdpi.com/article/10.3390/foods11142171/s1, Table S1: Related information on the macroporous adsorption resin used in this work, Table S2: Related information on the activated carbons used in this work (all the activated carbons are in powder form), Table S3: Related information on the ion exchange resins and fiber used in this work.
