# Supplementary material for: Effective Adsorption of Colorants from Sugarcane Juice by Bagasse-Based Biochar-Hydroxyapatite Composite
Source: Foods. 2022 Jul 21;11(14):2171. doi: 10.3390/foods11142171 (PMC9322621; doi:10.3390/foods11142171)
Supplement: Supplementary file 1 [file foods-11-02171-s001.zip › foods-1803919-supplementary.pdf]

foods

MDPI

Supplementary Materials

Effective Adsorption of Colorants from Sugarcane Juice by Bagasse-Based Biochar-Hydroxyapatite Composite

Cheng Wang [1], Mengying Luo [1], Caifeng Xie [1,2,3], Kai Li [1,2,3], Fangxue Hang [1,2,3,*], Changrong Shi [4,5,*] and William O.S. Doherty [4,5]

1 College of Light Industry and Food Engineering, Guangxi University, Nanning 530004, China; jonas_cheng_w@163.com (C.W.); luomengyinggxu@163.com (M.L.); fcx11@163.com (C.X.); gxlikai@gxu.edu.cn (K.L.)
2 Provincial and Ministerial Collaborative Innovation Center for Sugar Industry, Nanning 530004, China
3 Engineering Research Center for Sugar Industry and Comprehensive Utilization, Ministry of Education, Nanning 530004, China
4 Centre for Agriculture and the Bioeconomy, Faculty of Science, Queensland University of Technology, Brisbane, QLD 4000, Australia; w.doherty@qut.edu.au
5 School of Mechanical, Medical and Process Engineering, Faculty of Engineering, Queensland University of Technology, Brisbane, QLD 4000, Australia
* Correspondence: hangfx@163.com (F.H.); c.shi@qut.edu.au (C.S.)

Table S1. Related information on the macroporous adsorption resin used in this work.

Type	Manufacturer
D101	Tianjing Guangfu biochemical Co., Ltd

Table S2. Related information on the activated carbons used in this work (all the activated carbons are in powder form).

Number	Type	Manufacturer
1	Hongsheng	Dongguan Hongsheng Carbon Co., Ltd
2	Macklin	Shanghai Macklin Biochemical Co., Ltd
3	Sinopharm	Sinopharm Chemical Reagent Co., Ltd
4	Guanghua	Guangdong Guanghua Sci-Tech Co., Ltd

Table S3. Related information on the ion exchange resins and fiber used in this work.

Number	Type	Manufacturer
1	A722	Shanghai Macklin Biochemical Co., Ltd
2	D201	Beijing Solarbio Biochemical Co., Ltd
3	D301	Beijing Solarbio Biochemical Co., Ltd
4	IEF	Luoyang Xianrui Technology Co., Ltd
